# Operational analysis of the national sickle cell screening programme in the Republic of Uganda

**DOI:** 10.4102/ajlm.v10i1.1303

**Published:** 2021-08-12

**Authors:** Arielle G. Hernandez, Charles Kiyaga, Thad A. Howard, Isaac Ssewanyana, Grace Ndeezi, Jane R. Aceng, Russell E. Ware

**Affiliations:** 1 Division of Hematology, Cincinnati Children’s Hospital Medical Center, Cincinnati, Ohio, United States; 2 Global Health Center, Cincinnati Children’s Hospital Medical Center, Cincinnati, Ohio, United States; 3 Department of Epidemiology, Human Genetics and Environmental Sciences, School of Public Health, University of Texas Health Science Center, Houston, Texas, United States; 4 Central Public Health Laboratories, Ministry of Health, Kampala, Uganda; 5 Department of Paediatrics and Child Health, Makerere University College of Health Sciences, Kampala, Uganda; 6 Ministry of Health, Kampala, Uganda; 7 Department of Pediatrics, University of Cincinnati College of Medicine, Cincinnati, Ohio, United States

**Keywords:** Uganda, sickle cell disease, newborn screening, turn-around time, cost-effectiveness

## Abstract

**Background:**

Sickle cell anaemia is a common global life-threatening haematological disorder. Most affected births occur in sub-Saharan Africa where children usually go undiagnosed and die early in life. Uganda’s national sickle cell screening programme was developed in response to a 2014 sickle cell surveillance study that documented a high disease prevalence.

**Objective:**

This study describes the temporal and financial aspects of Uganda’s 2014–2019 sickle cell screening programme.

**Methods:**

National sickle cell screening data from Uganda’s Central Public Health Laboratories were used to calculate turn-around times (TATs) from sample collection to delivery, testing, and result reporting for blood samples collected from February 2014 to March 2019. The parameters affecting specific TATs were assessed. The exact programme expenditures were analysed to determine cost per test and per positive sickle cell disease case detected.

**Results:**

A total of 278 651 samples were analysed. The median TAT from sample collection to laboratory receipt was 8 days (interquartile range [IQR]: 6–12), receipt to testing was 3 days (IQR: 1–7), and testing to result reporting was 6 days (IQR: 3–12). Altogether, the sample continuum averaged 16 days (IQR: 11–24). Lower level healthcare facilities were associated with longer sample delivery TATs. Calendar months (January and December) and larger sample volumes impacted testing and result reporting TATs. The cost per test was $4.46 (United States dollars [USD]) and $483.74 USD per positive case detected.

**Conclusion:**

Uganda’s sickle cell screening programme is efficient and cost-effective. Universal newborn screening is the best strategy for detecting sickle cell anaemia in Uganda.

## Introduction

Sickle cell anaemia is a monogenic haematological disorder that manifests as a devastating systemic disease with high morbidity and early mortality. The pathophysiology begins in infancy with acute and life-threatening complications, which presents as increased susceptibility to infections, chronic haemolytic anaemia, and painful vaso-occlusive events.^1,2^ Sickle cell anaemia is the most prevalent haemoglobinopathy that impacts more than 20 million people worldwide, with an estimated 312 302 babies born with the disease each year.^3^ Over 75% of the world’s annual sickle cell anaemia births occur in sub-Saharan Africa where resources for providing early detection and care are most constrained,^3^ leading to the deaths of more than 500 affected children per day.^4^

The World Health Organization and other international groups have begun to recognise sickle cell anaemia as a major public health concern around the globe, but particularly in sub-Saharan Africa where there is a lack of government programmes to address this overwhelming and growing burden of disease.^5,6,7,8^ These organisations have identified the need to implement affordable evidence-based strategies that can be sustainably woven into existing healthcare systems, highlighting newborn screening as a priority intervention.^6^

Evidence from both high-resource and limited-resource countries have shown that newborn screening for sickle cell anaemia can significantly decrease morbidity and mortality by enabling early initiation of penicillin prophylaxis and pneumococcal immunisations as primary prevention.^9,10,11,12^ However, the accurate diagnosis of sickle cell anaemia in limited-resource settings still faces major challenges related to high equipment and reagent costs, education and training of healthcare providers, and appropriate medical interventions. Newborn sickle cell screening requires efficient laboratory methodologies and infrastructure for an early and accurate diagnosis to reduce preventable deaths among children born with sickle cell disease and to support the ultimate goal of universal screening of all babies across sub-Saharan Africa.

The Uganda Sickle Surveillance Study (US3) commenced in 2014 using a simple but innovative approach to screen children for the sickle cell trait and disease on a national level.^13^ Residual dried blood spot cards collected across the country as part of the Early Infant HIV Detection programme were tested. Isoelectric focusing (IEF) was conducted on about 100 000 samples over a cross-section of one year and found an overall high prevalence of sickle cell trait at 13.1% and disease at 0.7%, but with a disproportionate distribution across the country. Broader sickle cell screening has been initiated since 2015, strategically focused on the highest burden districts.

To expedite early detection and facilitate linkage to care for affected infants, it is crucial to identify where delays in diagnosis occur. Here, we describe a detailed operational analysis of the temporal and financial aspects of sickle cell screening in Uganda. We documented the turn-around times (TATs) for sickle cell screening, starting from sample collection to arrival, testing, and result reporting at the national centralised laboratory. To examine the cost implications of integrated sickle cell screening, we calculated the cost per test, based on exact expenditures from the US3, and made estimates of the cost-effectiveness of sickle cell screening in Uganda.

## Methods

### Ethical considerations

The initial US3 research proposal was approved by the School of Medicine Research Ethics Committee at Makerere University (No. 2012-138) in Kampala, Uganda, on 14 August 2012, with continuous annual renewal through August 2021. Approval from the Uganda National Council of Science and Technology was also completed. The proposal included an objective to conduct a cost-effectiveness analysis of the sample collection and testing process. As described in the US3 manuscript,^13^ the Uganda Ministry of Health waived informed consent for all samples tested as part of US3. Data integrity and patient privacy were assured through the use of de-identified specimen numbers maintained in a secured database.

### Integrated screening

US3 was conducted from February 2014 to March 2015 to determine the prevalence of sickle cell trait and disease in Uganda. A foundational goal of the study was building local sickle cell laboratory capacity within the Ministry of Health’s existing centralised laboratory infrastructure and determining the feasibility of the scale-up of newborn screening to the national level. At the Central Public Health Laboratories (CPHL) in Kampala, Uganda, a partnership with Cincinnati Children’s Hospital Medical Center led to the construction of a new sickle cell laboratory that was outfitted with IEF equipment, and local personnel were recruited and trained on a standardised haemoglobin electrophoresis protocol.

As previously described for the Early Infant HIV Detection programme, blood was collected from HIV-exposed infants and young children across the country using standard dried blood spot cards.^14^ Each health facility populated a dispatch form with demographic information and testing requests. Samples were transferred to the CPHL via the national sample transport system.^14^ In the US3, all samples requesting HIV testing were also queued for sickle cell testing; results were entered into the centralised database and disseminated back to health facilities and onward to caretakers. Following the US3, the dispatch form included an option for sickle cell testing only.

### Turn-around time analysis

The CPHL database collects laboratory data on all samples received and processed; data for this analysis were abstracted for children aged 0–24 months who underwent routine sickle cell screening over five years, from 01 February 2014 to 31 March 2019.

Inbound TAT was defined as the total number of days between sample collection at health facilities and the result dispatch from the CPHL back to healthcare providers. Three phases were defined within inbound TAT: (1) sample delivery phase TAT is the time between sample collection and receipt at the CPHL, (2) sample testing phase TAT is the time between receipt at the CPHL and date of testing, and (3) sample result reporting phase TAT is the time between testing and result dispatch from the CPHL. Outbound TAT, defined as the number of days between result dispatch and result receipt or subsequent action by the healthcare providers, was not assessed in this analysis because those data points are not currently collected by the CPHL.

Median TATs were calculated for inbound TAT and its three phases: sample delivery, testing, and result reporting using Stata statistical software (StataCorp LLC. 2019. Release 16. College Station, Texas, United States). The TAT calculation included weekends and holidays because the CPHL operates 24 hours a day, 7 days a week, 365 days a year. Summary statistics included medians, 25% and 75% interquartile ranges (IQR), and frequencies. Programme year 1 was the period of US3, followed by years 2 to 5 for every 12 months through March 2019. There were two distinct cohorts for sickle cell screening, based on how the sickle cell testing service was requested. The sickle/HIV co-testing cohort included all samples in the database that had both a sickle cell result and an HIV result. The sickle specific testing cohort included all samples that had a sickle cell result only.^15^

Phase-specific parameters were determined for sample delivery, testing, and result reporting TATs to assess different parts of the inbound sample continuum. For the sample delivery phase TAT, parameters included geographical region, health facility level, programme year, collection month, and testing cohort. For the sample testing phase TAT, parameters were programme year, testing month, and testing cohort. For the sample result reporting phase TAT, parameters were programme year, dispatch month, and testing cohort. Analysis of collection month was conducted using only year 5 data (01 April 2018 to 31 March 2019) to better understand recent temporal trends in TAT. Although there is no numerical value for laboratory efficiency or success, TAT is considered an indicator of laboratory performance, where the shorter the TAT the more efficient a laboratory is in producing results promptly.

### Cost analysis

The cost analysis of sickle cell screening was conducted by using Microsoft Excel (Microsoft, Corp., Redmond, Washington, United States) included the cost per test by IEF and cost per positive case detected. Costs were assessed by detailed analysis of all US3 expenditures over 13 months (01 February 2014 to 31 March 2015), using actual procurement documents and vendor price sheets for IEF equipment, reagents, and consumables, plus CPHL labour costs, including salary and other employment costs. Costs that were shared with other CPHL programmes and the health facilities were not included, such as the dried blood spot kits, sample transportation, local healthcare provider salaries, and screening-related training. Expenses incurred by Cincinnati Children’s Hospital Medical Center for travel and initial training of CPHL sickle cell laboratory personnel were also excluded. Cost for equipment, reagents, and consumables was reported in United States dollars (USD) according to actual costs using out-of-country and in-country vendors. For labour costs, annual salaries were expressed in Uganda shillings and converted to USD using the purchasing power parity exchange rate.^16^ The national sickle cell disease prevalence data for 2014–2019 and the calculated cost per test were used to generate the cost per sickle cell disease case detected by region within Uganda.

## Results

### Sickle cell testing turn-around time

Between 01 February 2014, and 31 March 2019, a total of 324 356 samples were collected and tested at the CPHL. Samples were excluded from analysis if they were outside the study date range, beyond 24 months of age, or collected and tested outside of routine sickle cell screening. The number of samples included in the final analysis was 278 651. In the subsequent analysis, only samples with present and plausible dates for collection, receipt, testing, or result dispatch were included depending on the TAT being calculated. Accordingly, there were 265 766 samples included in the inbound TAT calculation, 276 521 samples for sample delivery TAT, 263 417 samples for sample testing TAT, and 267 325 samples for sample result reporting TAT.

The overall median inbound TAT, from sample collection to result dispatch, was 16 days (IQR: 11–24) (Table 1). Most of the samples (*n* = 228 684, ~86%) did not exceed an inbound TAT of one month. Programme year 1 had the largest sample volume (*n* = 90 872), followed by almost 70 000 samples in year 4. The South Western region had the shortest median inbound TAT of 13 days (IQR: 9–20), while the Mid-Western region had the longest TAT of 18 days (IQR: 12–26). The sickle cell/HIV co-testing cohort had a slightly shorter inbound TAT (15 days) than the sickle specific cohort (17 days).

**TABLE 1:** Characteristics of sickle cell samples tested in Uganda from 01 February 2014 to 31 March 2019.

Characteristics	Inbound samples†	Median inbound turn-around time	
		Median (days)	Interquartile range‡
**Programme year (*n* = 265 766)**			
Year 1	90 872	11	8–15
Year 2	8002	22	17–29
Year 3	56 144	20	15–27
Year 4	69 052	21	14–33
Year 5	41 696	15	11–21
**Region (*n* = 265 755)**			
Central 1	37 127	16	11–24
Central 2	42 188	17	12–25
East Central	21 550	17	12–26
Kampala	53 124	14	9–22
Mid-Eastern	1595	16	11–23
Mid-Northern	44 730	18	12–26
Mid-Western	19 959	15	11–22
Northeast	14 327	15	10–23
Southwestern	15 395	13	9–20
West Nile	5760	15	10–23
**Testing cohort (*n* = 265 708)**			
Sickle cell/HIV co-testing	209 242	15	10–23
Sickle specific testing	56 466	17	12–25

Note: Inbound samples = 265 766; median inbound TAT - median (days) = 16; median inbound TAT - interquartile range = 11–24.

† , 12 885 samples did not have available dates for collection and dispatch to calculate inbound turn-around time;

‡ , 25% – 75% interquartile range.

For the sample delivery phase, the median TAT was 8 days (IQR: 6–12). Parameters affecting sample delivery TAT included the region of the requesting hospital and the level of healthcare provided (Table 2). The Kampala region, where the CPHL is located, had the shortest median sample delivery time of 6 days (IQR: 4–9), while six regions had a median sample delivery time of 9 days (Table 2). Regional referral hospitals had a shorter median sample delivery TAT of 7 days (IQR: 5–9) compared to the lower level health centres II, III, and IV. The collection month of December 2018 had the longest median TAT of 10 days (IQR: 5–18) compared to other collection months.

**TABLE 2:** Sample delivery phase turn-around time for sickle cell samples collected in Uganda, by parameter, February 2014 to March 2019.

Parameter	Samples collected†	Sample delivery turn-around time	
		Median (days)	IQR‡
**Region (*n* = 276 518)**			
Central 1	38 695	8	6–13
Central 2	43 494	9	7–13
East Central	22 464	9	6–13
Kampala	55 103	6	4–9
Mid-Eastern	12 117	9	6–13
Mid-Northern	46 333	9	6–13
Mid-Western	21 044	9	7–13
Northeast	14 878	7	5–11
Southwestern	16 335	9	6–14
West Nile	6047	8	6–12
**Health facility level (*n* = 275 894)**			
Regional Referral Hospital	17 895	7	5–9
Health Center IV	47 033	8	6–13
Health Center III	93 816	9	6–14
Health Center II	13 675	9	6–13
Hospital	72 336	6	6–13
Special Clinic	31 139	6	4–9
**Programme year (*n* = 276 521)**			
Year 1	97 782	8	6–12
Year 2	11 414	9	6–14
Year 3	56 203	8	6–12
Year 4	69 100	9	6–13
Year 5	42 022	8	5–12
**Year 5 collection month (*n* = 40 649)**			
April 2018	5422	8	4–11
May 2018	5163	7	3–10
June 2018	3263	8	5–12
July 2018	3394	7	4–11
August 2018	3361	8	5–12
September 2018	3240	9	6–13
October 2018	3371	8	5–12
November 2018	3094	8	5–12
December 2018	2309	10	5–18
January 2019	3159	8	5–11
February 2019	2825	9	5–13
March 2019	2048	6	3–9
**Testing cohort (*n* = 276 457)**			
Sickle/HIV co-testing	219 156	8	6–12
Sickle specific testing	57 301	9	6–13

*n* = 276 521.

IQR, interquartile range.

† , 2130 samples did not have available dates for when collected and received to calculate sample delivery turn-around time;

‡ , 25% – 75% interquartile range.

Once the sample arrived at the CPHL, the median sample testing TAT was 3 days (IQR: 1–7) and for sample result reporting TAT, it was 6 days (IQR: 3–12). For sample testing phase TAT, programme year 4 had the longest median TAT of 7 days (IQR: 4–14) (Table 3). Median sample testing and sample result reporting TATs were highest in the months of December 2018 and January 2019 (Table 3 and Table 4). The two testing cohorts had similar median sample testing and sample result reporting TATs.

**TABLE 3:** Sample testing phase turn-around time for sickle cell samples collected in Uganda, by parameter, February 2014 to March 2019.

Parameter	Samples tested†	Sample testing turn-around time	
		Median (days)	IQR‡
**Programme year (*n* = 263 417)**			
Year 1	94 184	0	0–1
Year 2	2157	4	2–5
Year 3	55 844	5	3–7
Year 4	69 377	7	4–14
Year 5	41 855	6	4–10
**Year 5 testing month (*n* = 42 201)**			
April 2018	6157	4	3–6
May 2018	5682	5	4–6
June 2018	3443	4	2–6
July 2018	3429	5	3–6
August 2018	3436	5	4–7
September 2018	3014	4	3–6
October 2018	3365	5	4–8
November 2018	3066	6	5–10
December 2018	1706	12	8–13
January 2019	3516	13	9–20
February 2019	2604	11	6–13
March 2019	2783	10	8, 15
**Testing cohort (*n* = 263 400)**			
Sickle cell/HIV co-testing	208 210	3	0–8
Sickle specific testing	55 190	4	2–6

*n* = 263 417.

IQR, interquartile range.

† , 15 234 samples did not have available dates for when received and tested to calculate sample testing turn-around time;

‡ , 25% – 75% interquartile range.

**TABLE 4:** Sample result reporting phase turn-around time for sickle cell samples collected in Uganda, by parameter, February 2014 to March 2019.

Parameter	Samples dispatched†	Sample result reporting turn-around time	
		Median (days)	IQR‡
**Programme year (*n* = 267 325)**			
Year 1	91 645	2	1–3
Year 2	8002	12	8–17
Year 3	56 381	10	7–15
Year 4	69 437	11	6–23
Year 5	41 860	6	4–10
**Year 5 dispatch month (*n* = 42 474)**			
April 2018	6319	5	3–6
May 2018	5720	5	4–7
June 2018	3441	5	3–6
July 2018	3455	5	3–6
August 2018	3440	5	4–7
September 2018	3056	4	3–7
October 2018	3363	5	4–8
November 2018	3072	6	5–10
December 2018	1704	12	8–13
January 2019	3517	13	9–20
February 2019	2605	11	6–13
March 2019	2782	10	8–15
**Testing cohort (*n* = 267 265)**			
Sickle cell/HIV co-testing	210 425	6	2–12
Sickle specific testing	56 840	6	4–12

*n* = 267 325.

IQR, interquartile range.

† , 11 326 samples did not have available dates for when tested and dispatched to calculate sample result reporting turn-around time;

‡ , 25% – 75% interquartile range.

### Sickle cell screening costs

Inputs included in the cost analyses were exact direct expenditures for equipment, reagents, consumables, and labour for 99 243 US3 samples tested by IEF from a dried blood spot at the CPHL over 13 months (Table 5). The total equipment costs were annualised and calculated at $0.94 USD per test. Reagent costs and consumables were calculated at $1.04 USD and $0.15 USD per test. The annual salary for laboratory personnel included gross pay, National Social Security Fund 10% monthly tax, customary ‘13th month’ end-of-year compensation, and medical insurance per standard Ministry of Health employee contracts. Labour costs were $2.33 USD per test, for an overall cost per test of $4.46 USD. Personnel costs made up more than half of the cost per test total at 52%, followed by reagents (23%), equipment (21%), and consumables (3%).

**TABLE 5:** Costs for sickle cell screening and testing for 99 243 children in Uganda from the US3 project over 13 months, February 2014 to March 2015.

Cost category	Item	Quantity	Cost (USD)¶
Equipment	Electrophoresis unit	4	$21 600.00
	Water bath	-	$5800.00
	Power supply	2	$11 600.00
	Rocking platform	-	$1200.00
	Gel dryer	-	$670.00
	Puncher	-	$15 600.00
	Puncher computer	-	$1900.00
	Puncher workstation	-	$1900.00
	Puncher printer	-	$165.00
	Glow box	-	$964.00
	Replacement electrode	6	$3528.00
	Freezer	-	$4400.00
	Refrigerator	-	$6900.00
	Distiller	-	$3145.00
	Value-added tax†	-	$14 340.96
	Equipment total	-	$93 712.96
	Equipment cost per test total	-	$0.94‡
Reagents (cost per test)	RESOLVE haemoglobin kit	-	$0.83
	HbFASC control kit	-	$0.06
	JB-2 stain solution kit	-	$0.12
	Trichloroacetic acid	-	$0.03
	Reagent cost per test total	-	$1.04
Consumables (cost per test)	96-well plates	-	$0.07
	Gloves	-	$0.08
	Pipette tips	-	$0.01
	Consumables cost per test total	-	$0.15
Labour (annual salary)	Laboratory manager	-	$50 056.01
	Laboratory technician	2	$86 763.74
	Laboratory assistant	-	$20 022.40
	Data officer	-	$33 370.67
	Data clerk	-	$20 022.40
	Personnel medical insurance	-	$20 945.82
	Personnel total	-	$231 181.05
	Labour cost per test total	-	2.33§

**Cost per test total**	**-**	**-**	**$4.46**

US3, Uganda Sickle Surveillance Study; USD, United States dollars.

† , Value-added tax for imported equipment;

‡ , Equipment total/99 243 children screened;

§ , Personnel total/99 243 children screened;

¶ , 2015 purchasing power parity exchange rate ($1071.30) applied for conversion from Ugandan shillings to USD.

In Uganda, the highest burden of sickle cell disease is observed in the East Central (1.6%) and Mid-Northern (1.5%) regions (Table 6). Using the calculated total cost per test and national prevalence data, the cost per sickle cell disease case detected was $483.74 USD (Table 6). When stratified by region, however, the cost per positive case detected ranged widely from $278.07 USD in the East Central region to $2607.19 USD in the South Western region.

**TABLE 6:** Cost per positive sickle cell disease case in Uganda, by region from February 2014 to March 2019.

Region	Sickle cell disease cases	Total screened	Prevalence	Cost per positive case (USD)
Central 1	214	36 173	0.0059	$753.89
Central 2	217	23 872	0.0091	$490.64
East Central	308	19 203	0.0160	$278.07
Kampala	296	40 635	0.0073	$612.27
Mid-Eastern	147	11 109	0.0132	$337.05
Mid-Northern	527	35 671	0.0148	$301.88
Mid-Western	119	19 686	0.0060	$737.81
Northeast	148	12 151	0.0122	$366.17
South Western	28	16 368	0.0017	$2607.19
West Nile	32	5959	0.0054	$830.54

**Total**	**2036**	**220 827**	**0.0092**	**$483.74**

USD, United States dollar.

## Discussion

For any newborn screening programme, reduction of sample TAT is an important intervention to ensure that poor health outcomes or preventable deaths are not the results of laboratory or operational delays. Identification of the specific barriers related to longer TAT and implementation of measures to mitigate those causes are essential to strengthen screening programmes.

**FIGURE 1:**
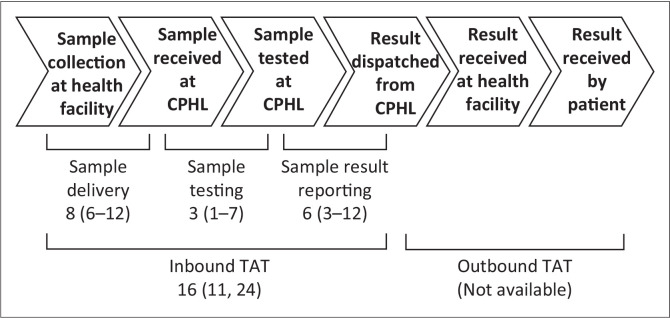
Uganda Central Public Health Laboratories sample continuum and turn-around time phases, with results shown as the median in days (25% – 75% IQR) from February 2014 to March 2019. CPHL, Central Public Health Laboratories; TAT, turn-around time; IQR, interquantile range.

In this first detailed operational analysis of TAT for sickle cell screening in Uganda, the total duration between sample collection at the health facility to the dispatch of results from the CPHL averaged 16 days (Figure 1). Multiple parameters affected this time interval, including the health facility level, programme year, collection month, and testing cohort. However, the vast majority (~86%) had results sent by CPHL back to the local healthcare facilities within one month of receipt (Table 1), which we propose as the maximal TAT threshold before putting sickle cell disease patients at risk by delaying diagnosis and treatment. Penicillin prophylaxis and pneumococcal immunisations are expected to be administered by three months of age, and these TAT results are sufficient to meet those goals.^9,10,11,12^

For the Uganda sickle cell screening programme, sample delivery accounted for most of the inbound TAT (Figure 1). Lower health facilities likely have greater barriers to timely delivery, such as delayed collection of their samples for transport (Table 2). Within each district, hub motorbike riders perform a weekly pickup from the more rural locations of health centres II, III, and IV in comparison to regional referral hospitals or district hospitals that experience daily pickup. It is also possible that samples are not readily available for transport at the time the hub motorbike riders come for collection, or some facilities are missed due to inclement weather or mechanical problems. To address these issues, a twice-weekly sample pickup with validation measures, such as timestamps, should be put into place. This should also be supplemented with ongoing training regarding sample preparation and the importance of timely sample transport for all personnel involved in the process.

After sample receipt at the CPHL, sample testing was relatively rapid with an average of only 3 days (Figure 1). This phase involves the routing of samples for sickle cell testing to the laboratory, punching and eluting of dried blood spots, testing by IEF by highly trained personnel, and entering of results into database. Based on the short TAT, this internal process can be deemed efficient. However, when looking at the parameter of the programme year, it does appear that longer TAT in this phase is associated with periods of increased sample volumes (Table 3). This may be the cause of reagent stock out or point to the need for more cross-training of CPHL personnel on the IEF procedure to support the sickle cell laboratory. As the programme expands toward the eventual goal of universal newborn screening, more human resources will be necessary to handle the increased number of samples.

Following sample testing, result reporting took an average of 6 days (Figure 1). Altogether, the time that samples spent at the CPHL was prolonged. Delays with the dispatch of results could be sample retesting or stock out of printing supplies. It was also observed that the testing and dispatch months of December 2018 and January 2019 had distinctly longer TATs (Table 4). This may be linked to personnel leave due to the holidays, thus increasing or staggering support in the sickle cell laboratory during this time could help avoid lengthy delays.

In a previous study by Kiyaga et al. at the Uganda CPHL regarding TAT of Early Infant HIV Detection screening, the average sample delivery TAT was 12 days, sample processing was 2 days, and the overall average TAT from sample collection to reporting to the patient was 26 days.^14^ As part of this study, short message service printers were piloted to allow test results to be transmitted immediately to healthcare facilities after testing, which further reduced the overall TAT to 14 days.^14^ In the United States, the National Newborn Screening and Global Resource Center reported newborn bloodspot screening TAT to be within 10 to 14 days from sample collection, with considerable variation by state.^17^ To overcome these differences in state programmes, the United States Department of Health and Human Services Advisory Committee on Heritable Disorders in Newborns and Children recently set out updated newborn screening timeliness goals specifying that tests should be completed within 7 days of birth.^18^ In our study, we found that TAT for sickle cell screening was shorter in all inbound phases compared to what was previously reported for Uganda’s Early Infant HIV Detection programme, and exhibited overall improved time efficiency for the entire CPHL sample continuum (median 16 days vs 26 days). The current programme is approaching a comparable timeframe to that of the long-standing United States newborn sickle cell screening programme and will likely continue to evolve and improve over time.

Information on outbound TAT, which includes time to family notification and matriculation to care, could not be examined in this study, because those data are currently not collected by the CPHL. However, with greater visibility and a push toward newborn screening in sub-Saharan African countries, steered by the new American Society of Hematology African Screening Consortium,^19^ outbound TAT and individual patient follow-up data will be coveted information. We predict that TAT will greatly impact healthcare provider and patient utilisation of sickle cell screening, as well as overall satisfaction with the programme in Uganda. Most importantly, prolonged TAT very likely postpones life-saving treatments for patients with the disease, if the diagnosis is not established and communicated promptly. Also, delayed result reporting can lead to affected infants being lost to follow-up and may require a replacement test. Improving TAT helps ensure that caretakers collect their results on time and minimises the need for inefficient and expensive repeat testing. Ultimately, making improvements in all TAT phases for sickle cell screening is essential to optimise infant health outcomes, cost-effectiveness, and screening satisfaction for healthcare providers and patients in Uganda.

A major strength of this analysis was the complete national representative long-term data from the CPHLs centralised database, which allowed us to investigate previously undocumented parameters that affect sickle cell testing TAT in Uganda. Limitations include the access to only inbound TAT data, which limits our ability to connect our findings to patient indicators such as notification and receipt of sickle cell results, followed by care and management of affected patients. Another limitation is the lack of recommendations for sickle cell testing TAT by the Uganda Ministry of Health, other regional programmes, or by international bodies such as the World Health Organization for a comparison of efficiency. However, our data provided the first detailed time analysis of sickle cell screening in sub-Saharan Africa, to enable monitoring and evaluation of future interventions and other programmes.

Analysis of the direct costs of a sickle cell screening programme in Uganda showed that the cost per test by IEF using dried blood spots was $4.46 USD. In Angola, the cost per infant screened in a newborn screening programme that used IEF was $15.36 USD, or $7.42 USD when considering just the inputs of laboratory personnel, reagents, consumables, and equipment as in our study.^20^ The cost of the screening test for sickle cell disease performed by IEF in the United States was $2.29 USD and approximately $4.50 USD (reported as £3.51) in the United Kingdom.^21,22^ The United States and United Kingdom have addressed the cost‐effectiveness of newborn screening for sickle cell disease for universal and targeted programmes. Both nations have justified the greater cost incurred by screening every newborn for sickle cell disease, because it identifies all infants with the disease, prevents more deaths, and has demonstrated better outcomes for patients.^21,22,23^ In Africa, modelling simulations have found universal newborn sickle cell screening to be extremely cost-effective, especially in countries with a high disease prevalence of 0.2% – 0.5%.^24,25^ Yet, no federal newborn sickle cell screening currently exists in any country in sub-Saharan Africa, despite the well-evidenced economic and humanitarian basis for such programmes.

In Uganda, the estimated costs per sickle cell disease case detected among the 10 regions varied from $278.07 USD in the East Central to nine times that in the South Western region ($2607.19 USD) (Table 6). These results show that newborn screening in regions with low sickle cell disease prevalence would result in a higher cost per positive case detected compared with screening focused in high-burden areas. However, because Uganda is a country with a high overall disease prevalence of 0.9%,^26^ universal newborn screening would still be the most economical and impactful public health strategy for sickle cell disease across the entire country.

### Limitations

This analysis only considered costs and numbers of cases detected for partial cost-effectiveness analysis. More extensive studies with patient follow-up data will be vital to provide evidence that screening is effective in reducing sickle cell morbidity and mortality, the benefits and harms of screening, and the long-term cost-effectiveness of a newborn sickle cell screening programme in Uganda.

### Conclusion

This study provides a contemporary and detailed description of the time and costs of sickle cell screening in Uganda. Analysis of the different phases of TAT highlights areas for improvement to reduce the number of samples with excessive delays, and to strengthen the overall integrated CPHL sample continuum. The lack of sickle cell testing guidelines limits our ability to compare these results to screening and care standards; however, this study documents that the CPHL centralised database can be used to accurately monitor and manage TAT for sickle cell screening and can identify factors affecting TAT at different phases to prompt targeted improvements. This study also shows that sickle cell testing by IEF is provided at under $5.00 USD, and the strategy of universal newborn screening is cost-effective to save and improve the lives of thousands of individuals with sickle cell disease in Uganda.
